# A neuromorphic spiking neural network detects epileptic high frequency oscillations in the scalp EEG

**DOI:** 10.1038/s41598-022-05883-8

**Published:** 2022-02-02

**Authors:** Karla Burelo, Georgia Ramantani, Giacomo Indiveri, Johannes Sarnthein

**Affiliations:** 1 Klinik für Neurochirurgie, Universitätsspital und Universität Zürich, 8091 Zurich, Switzerland; 2 Institute of Neuroinformatics, University of Zurich and ETH Zurich, Zurich, Switzerland; 3 Neuropädiatrie, Universitäts-Kinderspital und Universität Zürich, Zurich, Switzerland; 4 Forschungszentrum für das Kind, Universitäts-Kinderspital Zürich, Zurich, Switzerland; 5 Zentrum für Neurowissenschaften Zürich, ETH und Universität Zürich, Zurich, Switzerland

**Keywords:** Epilepsy, Electroencephalography - EEG, Biomedical engineering

## Abstract

Interictal High Frequency Oscillations (HFO) are measurable in scalp EEG. This development has aroused interest in investigating their potential as biomarkers of epileptogenesis, seizure propensity, disease severity, and treatment response. The demand for therapy monitoring in epilepsy has kindled interest in compact wearable electronic devices for long-term EEG recording. Spiking neural networks (SNN) have emerged as optimal architectures for embedding in compact low-power signal processing hardware. We analyzed 20 scalp EEG recordings from 11 pediatric focal lesional epilepsy patients. We designed a custom SNN to detect events of interest (EoI) in the 80–250 Hz ripple band and reject artifacts in the 500–900 Hz band. We identified the optimal SNN parameters to detect EoI and reject artifacts automatically. The occurrence of HFO thus detected was associated with active epilepsy with 80% accuracy. The HFO rate mirrored the decrease in seizure frequency in 8 patients (*p* = 0.0047). Overall, the HFO rate correlated with seizure frequency (rho = 0.90 CI [0.75 0.96], *p* < 0.0001, Spearman’s correlation). The fully automated SNN detected clinically relevant HFO in the scalp EEG. This study is a further step towards non-invasive epilepsy monitoring with a low-power wearable device.

## Introduction

### Epilepsy and EEG biomarkers

Epilepsy is one of the most common neurological disorders globally. The standard initial treatment for epilepsy is anti-seizure medication (ASM), resulting in seizure freedom in 60–70% of epilepsy patients, while epilepsy surgery may be an effective treatment option for a subset of the remaining patients with focal lesional epilepsy^1–8^.

Monitoring the disease state in epilepsy is the key to assessing the efficacy of ASM or epilepsy surgery in achieving seizure control and identifying periods with low or high seizure propensity that will require therapy adjustments over time. Therapy monitoring is crucial for facilitating personalized medicine, thus improving not only seizure outcomes but also the quality of life in patients with epilepsy. However, the current gold standard for assessing any therapeutic intervention in epilepsy is self-reported seizure frequency, i.e. seizure diaries that have often proven unreliable^9–11^. In EEG, the presence or absence of epileptiform potentials such as spikes is a sensitive and easily accessible marker of epileptogenicity. However, epileptiform potentials lack a stable correlation with disease activity, undermining their reliability as a biomarker to monitor treatment response^12^. Thus, other reliable and practicable biomarkers are urgently needed.

### HFO in the scalp EEG

In recent years, interictal High Frequency Oscillations (HFO), initially recorded directly from epileptogenic brain tissue in intracranial EEG (iEEG), have been identified as a reliable biomarker of epileptogenicity^13^. Evidence has lately accumulated that epileptic HFO is also measurable by non-invasive scalp EEG^14–24^.

HFO are currently investigated as potential biomarkers of epileptogenesis, seizure propensity, disease severity, and treatment response (for reviews see^13^ and^25^). Given the widespread access to non-invasive EEG, scalp HFO may have the potential for clinical assessment in a broad population of patients affected by epilepsy.

Assessment of the disease state with a wearable device is considered advantageous since epilepsy is an unpredictable disease^26–28^. However, a conventional device for HFO detection requires high sample rate input. By nature, this feature demands expensive calculations and large data storage/transfer, thus increasing energy consumption and size and precluding implementation in a wearable device.

### Neuromorphic devices

The large amount of sensory data recorded by a wearable device calls for the development of low-power embedded "edge-computing" technologies that can process the signals being measured locally without requiring bulky computers, internet connection, or cloud servers. When developing a wearable sensory-processing device, neuromorphic engineering represents a promising technology.

Neuromorphic electronic circuits are a class of hybrid analog/digital circuits that implement hardware models of biological systems^29,30^. They carry out computation by emulating the dynamics of real neurons and synapses, configured as small spiking neuronal networks (SNNs). The styles of computation used in neuromorphic circuits are fundamentally different from those used by conventional computers. Neuromorphic architectures provide massively parallel arrays of computing elements, exploit redundancy to achieve fault tolerance, and emulate the neural style of computation. In this way, neuromorphic systems can exploit to the fullest potential the features of advanced scaled Very Large Scale Integrated (VLSI) processes and future emerging technologies, naturally coping with the problems that characterize them, such as device inhomogeneity and imperfections. Consequently, neuromorphic networks can be implemented in compact VLSI devices to perform real-time computation with low energy consumption^8,29,31^.

### Outline

In a previous study^14^, we analyzed pediatric scalp EEG using our Spectrum HFO detector and showed that (1) the location of the highest HFO rate in scalp EEG corresponds to the location of the highest HFO rates in electrocorticography (ECoG), (2) scalp HFO rates correlate with seizure frequency (R^2^ = 0.80), and (3) scalp HFO rates are higher in patients with active epilepsy (accuracy = 84%) and decrease in response to successful surgical treatment. In the current study, we re-analyzed this previously published dataset^14^ using an SNN detector, ideally suited for neuromorphic signal processing hardware. We extended a previously designed SNN that was proven effective in detecting HFO in the presurgical long-term intracranial EEG (iEEG)^8^ and the intraoperative ECoG^32^. Here, we tested whether the extended SNN can reliably detect clinically relevant HFO also in the pediatric scalp EEG.

## Methods

### Patients

We re-analyzed the scalp EEG recordings of children and adolescents with drug-resistant focal lesional epilepsy that underwent presurgical evaluation, resective epilepsy surgery, and postsurgical follow-up (median 45 months) in the University Children’s Hospital Zurich and had been considered for our previous study^14^. Twenty recordings from 11 patients (8 male) fulfilled the inclusion criteria of scalp EEG (1) recorded at a high sampling frequency (> 2000 Hz), (2) containing at least 10 min of NREM sleep, and (3) recorded at > 2 h from the most recent seizure. In all cases, we could form a clear hypothesis regarding the localization of the epileptogenic zone based on electro-clinical findings and the presence of an MR-visible lesion.

Seizure frequency, as a measure of epilepsy severity, was assessed by long-term video-EEG or seizure diaries at the time of the pre- or postsurgical EEG. Postsurgical seizure outcome was portrayed according to the ILAE scale. Epilepsy substrates were determined by histopathology. The data has been previously made available at https://gin.g-node.org/USZ_NCH/Scalp_EEG_HFO. All patients before surgery and patients with seizure recurrence after surgery (ILAE 2–5) were defined as having active epilepsy.

### EEG recordings

All patients underwent routine presurgical and postsurgical EEG using an 8-channel custom-made low-noise amplifier (LNA) with an input noise level of ~ 2.3 nV/√Hz, in addition to the commercial device. Given the limited number of available LNA channels, we connected four adjacent electrodes over the presumed epileptogenic zone and four homologous electrodes over the contralateral hemisphere. Impedances were typically around 1 kΩ. Data were acquired at 10 kHz and down-sampled to 2 kHz for further processing.

### Data selection and previous analysis

EEG was recorded while patients took afternoon naps. We selected NREM sleep intervals exclusively since HFO rates are higher during NREM than REM sleep^20,33^. Intervals with visible artifacts and channels with continuous interference were excluded from further analysis. The resulting data (mean duration of 27 ± 13 min per patient) was divided into 5-min intervals.

The data considered in the present study has been previously analyzed using the Spectrum detector^14^. The Spectrum detector has also been used to detect clinically relevant HFO in other data sets in order to predict postsurgical seizure outcome^14,34–39^. While we present previous results of the Spectrum detector for comparison, the main focus of this study is the detection of clinically relevant HFO with the SNN, as presented in detail below.

### HFO detection with the SNN

**Figure 1:**
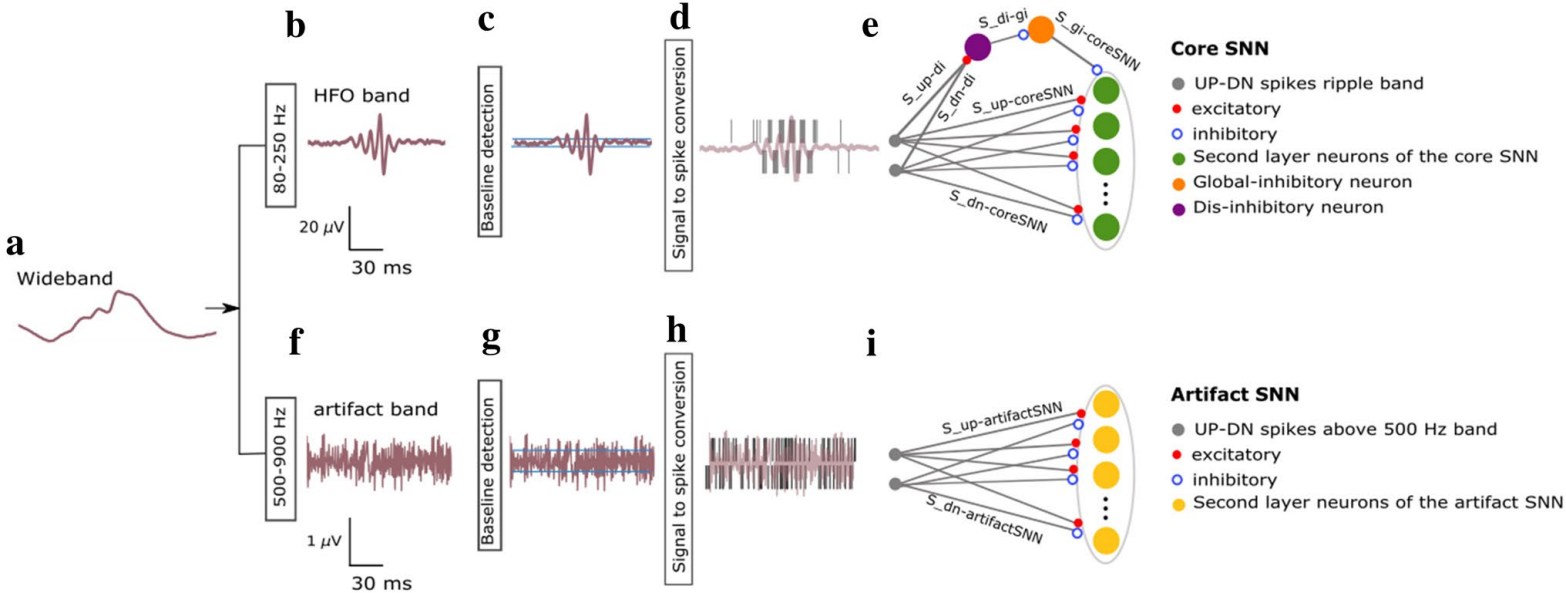
HFO detection scheme. The HFO detector performs three preprocessing stages before sending the data to the SNN. The preprocessing stages are filtering, baseline detection, and converting the analog signal into spikes. (**a**) The EEG signal is filtered in the ripple band (80–250 Hz) (**b**) and 500–900 Hz above the fast ripple band (**f**). (**c**,**g**) In the baseline detection stage, the background noise of each signal is used to set the signal-to-spike threshold. (**d**,**h**) The signal-to-spike conversion algorithm converts each analog signal into two streams of digital outputs: UP and DN spikes. (**e**) The core SNN architecture for HFO detection consists of input neurons (grey) receiving the input UP-DN spikes from the filtered signal in the ripple band. These inputs project to a second layer of neurons (green) and to a dis-inhibitory neuron (purple). This neuron projects inhibitory synapses to a global-inhibitory neuron (orange), which is continuously inhibiting the second layer neurons. The synapses of the projections are excitatory (positive, red) or inhibitory (negative, blue). The role of the interneuron and the inhibitory neuron is to avoid the false detection of sharp transients^32^. When enough neurons in this layer are activated, they elicit spikes, indicating an EoI. (**i**) The artifact rejection stage of the SNN consists of input neurons (grey) receiving the input UP-DN spikes from the filtered signal above 500 Hz. These inputs project to a second layer of neurons (yellow). When enough neurons in this layer are activated, they elicit spikes, indicating an artifact's presence. When the neurons in the core SNN (green) are active simultaneously as the neurons in the artifact SNN (yellow), the EoI is rejected. Conversely, when the neurons in the core SNN (green) are active, and the neurons in the artifact SNN (yellow) are silent, the EEG SNN classifies the EoI as HFO.

The SNN model used is a two-layer network comprising leaky integrate-and-fire (LIF) neurons and synapses with biologically realistic temporal dynamics. To take into account the variability introduced by the neuromorphic analog circuits used to implement the SNN, the hyper-parameters of both synapses and neurons (such as time constant or synaptic weights) were drawn randomly from a normal distribution with a coefficient of variation parameters matched to those measured from the electronic circuits (see^8^ for details). As the first step in our HFO detection pipeline, we used the EEG front-end signal-processing stages described in our previous work^8,32^. In summary, the wideband EEG (Fig. 1a) was filtered in the 80–250 Hz ripple band using 2nd order Butterworth filters (Fig. 1b), which are a good approximation of the Tow-Thomas architectures in a hardware implementation of the HFO detector (hardware SNN)^8,40,41^. We next calculated the background noise in the filtered signal to define a baseline amplitude that had to be exceeded by a putative HFO event (Fig. 1c). The threshold for converting the analog signal into spikes was set at 30% of the calculated baseline amplitude. The signal-to-spike conversion algorithm simulated the operations of an asynchronous delta modulator (ADM) circuit (Fig. 1d)^8,42,43^. At any given time, the ADM algorithm checks if the signal has increased or decreased above a threshold (defined by the user) and then accordingly generates either a positive (UP) or a negative (DN) spike. After generating a spike, the ADM algorithm takes the amplitude value at that time as the new baseline. The resulting UP-DN spike trains are then provided as inputs to the SNN. Compared to the analysis of iEEG^8^ and ECoG^32^, we had to reduce the signal-to-spike threshold of the ADM because the scalp EEG has a smaller signal amplitude than ECoG and iEEG.

The HFO detection stage of the network consisted of input neurons receiving the UP-DN spikes and a second layer of neurons (Fig. 1e). The projections to the second layer of neurons were via excitatory synapses for UP spikes and inhibitory ones for DN spikes. The synaptic circuits implement biologically plausible dynamics, producing output currents in response to input spikes that decay exponentially. The response amplitude and decay time are set by the synaptic weight parameter and the time constant, respectively. For these connections, we used the sets of synaptic time constant and weight parameters that were previously optimized for HFO detection in iEEG^8^. Additionally, the SNN included a global-inhibitory neuron that constantly suppressed the activity of the neurons in the second layer to avoid their responding to fast transients in the ripple band and a dis-inhibitory neuron that stopped this inhibition to allow the second layer of neurons to respond to an HFO^32^. The parameters for the global-inhibitory and dis-inhibitory neurons in the SNN had been found heuristically by analyzing HFO and sharp transients in our previous study with intraoperative ECoG^32^.

We then used this SNN (the core SNN) to detect potential HFO. The synaptic parameters of the SNN were the same in all three data types: ECoG, iEEG, and scalp EEG. Spikes in the second layer of neurons in the core SNN were used to mark an Event of Interest (EoI). Any spike within a 15 ms window indicated an EoI. Consecutive windows containing spikes were concatenated to form a continuous EoI. HFO detection was performed independently for each bipolar channel of the presurgical and postsurgical EEG. There was neither visual inspection nor manual rejection of events in this fully automated algorithm.

We used the Python SNN simulator Brian2^44^, the custom toolbox Teili^45^, and the parameters in Table 1 to simulate an SNN that matches the behavior of the neuromorphic circuits of the hardware SNN^8^.

**Table 1:** EEG SNN parameters.

Connection	Name	Connection strength (fA)	Polarity	Time constant τ (ms)
Input above 500 Hz UP spikes to second layer artifact SNN	S_up-artifactSNN_	[7–14]	exc	[3–6]
Input above 500 Hz DN spikes to second layer artifact SNN	S_dn- artifactSNN_	[7–14]	inh	S_up-artifactSNN_—[0.1–1]
Input ripple UP spikes to second layer core SNN	S_up-coreSNN_	[7–14]	exc	[3–6]
Input ripple DN spikes to second layer core SNN	S_dn- coreSNN_	[7–14]	inh	S_up-coreSNN_—[0.1–1]
Input UP spikes to dis-inhibitory neuron	S_up- di_	21	exc	5
Input DN spikes to dis-inhibitory neuron	S_dn- di_	21	exc	5
Dis-inhibitory neuron to global-inhibitory neuron	S_di-gi_	17.5	inh	20
Global-inhibitory neuron to second layer core SNN	S_gi- coreSNN_	21	inh	5

Synapse parameters of the EEG SNN detector. A connection between two neurons is characterized by the positive (excitatory, exc) or negative (inhibitory, inh) current in fA and the time constant.

The software simulations are compatible with the neuromorphic circuit properties. Since the circuits are based on a current-mode design, we used currents to set the network parameters. For example, the time constant for the synapse between the UP input and the dis-inhibitory neuron was set to 21 fA, which corresponds to a time constant of 5 ms (Table 1).

### Clinical validation of HFO

Automated HFO detection, visual validation, and analysis were performed blinded to clinical data. HFO were not used for clinical decision-making. In this study, we did not train the SNN with a predefined set of HFO (e.g., expert scoring) but rather validated the HFO detected by the SNN against the patient's disease state^46^. As the main focus of our study, we compared the output of the HFO detection to whether the epilepsy was active or not in that patient.

To test this association of HFO occurrence and active epilepsy, we first calculated the mean HFO rate in each electrode channel of the recordings of each patient by dividing the number of HFO detected in the channel by the duration of the recording. We included only the channels located on the hemisphere affected by epilepsy. We defined a rate threshold of 0.25 HFO/min, as previously computed using the HFO rates found by the Spectrum detector^14^, i.e., the recording was defined as showing HFO only if the HFO rate exceeded the rate threshold. We then defined as true positive (TP) an EEG showing HFO in patients with active epilepsy (all patients before surgery; patients with postoperative seizure outcome ILAE 2–5). We defined as false positive (FP) an EEG showing HFO in seizure-free patients after surgery (ILAE 1). We defined as false negative (FN) an EEG showing no HFO in patients with active epilepsy. We defined as true negative (TN) an EEG showing no HFO in seizure-free patients after surgery. The positive predictive value (PPV) was calculated as PPV = TP/(TP + FP), the negative predictive value (NPV) as NPV = TN/(TN + FN), the sensitivity = TP/(TP + FN), the specificity = TN/(TN + FP), and the accuracy = (TP + TN)/N, where N indicates the total number of recordings.

### Statistics

The correlation between HFO rate and seizure frequency was estimated using linear regression with ordinary least squares and Spearman’s correlation. We compared HFO rates between recordings with the Wilcoxon rank-sum and matched pair signed-rank tests. To test case-wise changes in HFO rate and seizure frequency between presurgical and postsurgical recordings, we used the χ^2^ test. Statistical significance was established at *p* < 0.05.

### Ethical considerations

The collection of patient data and the scientific analysis were approved and performed according to the guidelines and regulations of the ethics committee (Kantonale Ethikkommission Zürich, KEK-ZH PB-2016-02055), and all patients and their parents gave written informed consent.

## Results

### The SNN for HFO detection in scalp EEG

For our HFO detection pipeline we used the core SNN as implemented in our previous studies deriving from iEEG and ECoG recordings^8,32^, and augmented it by an additional SNN for the detection of artifacts in the scalp EEG. We refer to the resulting network architecture as EEG SNN (Fig. 1). The goal of the artifact detection SNN is to capture oscillations that may resemble an HFO, but should be rejected based on their high amplitude and frequency.

The core SNN receives input EEG in the 80–250 Hz ripple band. It consists of two input neurons that project the UP and DN spike trains to the second layer of neurons using excitatory and inhibitory synapses, respectively. In addition, the core SNN contains a global inhibitory neuron and a dis-inhibitory neuron that suppress HFO detection during fast transient artifacts in the ripple band. We view the output of the core SNN as EoI.

The high rate of fast transient artifacts in the scalp EEG (compared to iEEG and ECoG) required the design of an additional artifact detection SNN specifically for this study. The artifact detection SNN processes EEG data simultaneously to the core SNN to suppress HFO detection during fast transient artifacts in the 500–900 Hz band (Fig. 1i). The > 500 Hz band was chosen to avoid possible overlap with fast ripple HFO in the 250–500 Hz band. In this way, fast transient artifacts are detected but not confounded with fast ripple HFO that may be associated with clinically relevant ripple HFO^38,39^.

The artifact detection SNN used the same preprocessing stages as the core SNN, but the input signal was filtered in the 500–900 Hz frequency band (Fig. 1f,g,h). The input EEG was converted to UP and DN spikes projected to the second layer of neurons using dynamic synapses. The weights and time constants of the synapses were in the same range as those in the core SNN (Table 1). Any occurrence of a spike in the second layer neurons marked an artifact as the output of the artifact SNN.

**Figure 2:**
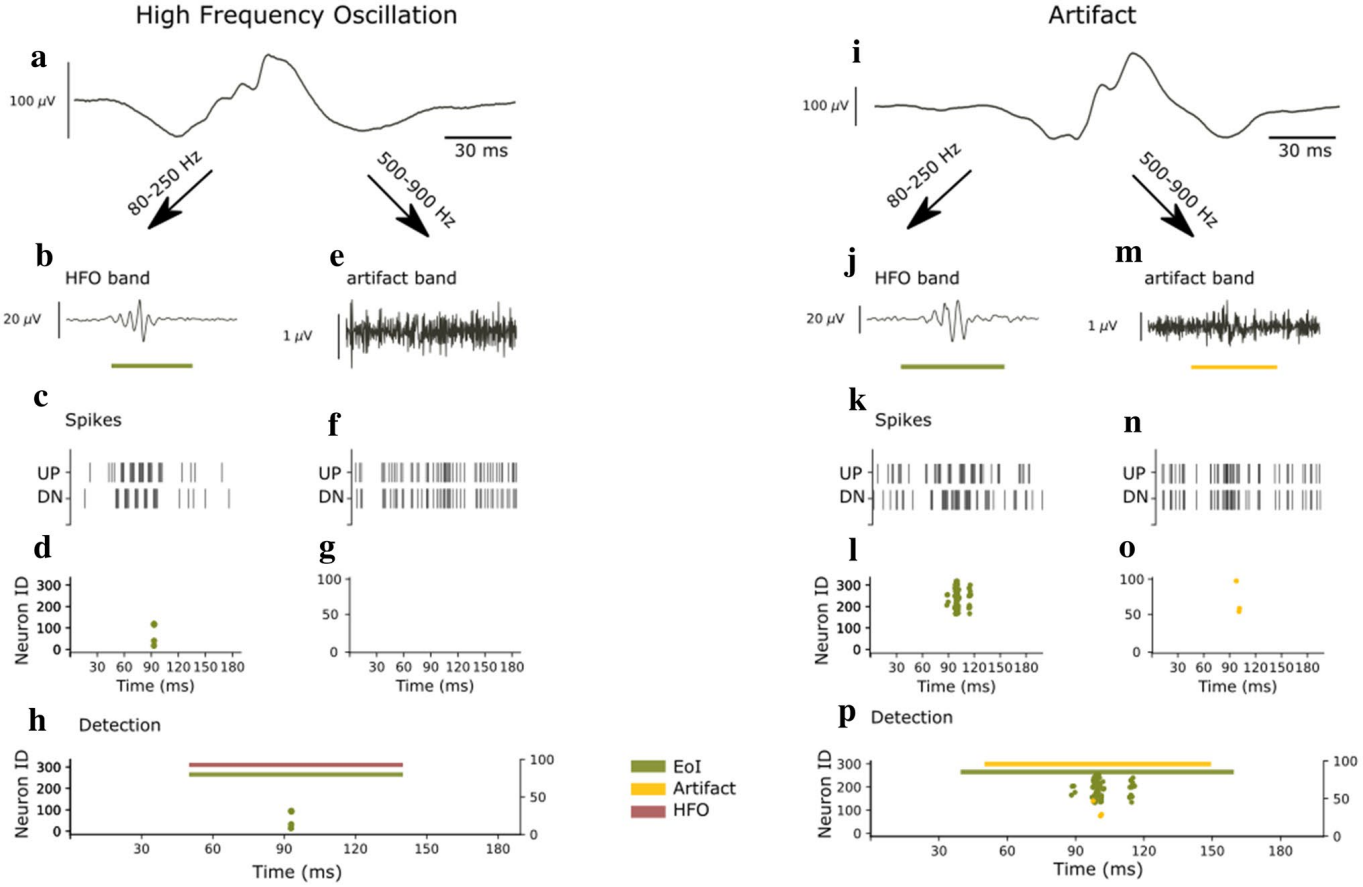
Example of a detected HFO and an artifact. The EEG SNN can distinguish if an EoI in the EEG is an HFO or an artifact. (**a**,**i**) EEG signal as recorded. (**b**,**j**) EEG signal filtered in the ripple band (80–250 Hz). (**e**,**m**) EEG signal filtered in the 500–900 Hz band. (**c**,**k**) Input spike trains to the core SNN. (**f**,**n**) Input spike trains to the artifact detection SNN. (**d**,**l**) Raster plot of the neurons in the second layer of the core SNN. (**g**,**o**) Raster plot of the neurons in the second layer of the artifact detection SNN. (**h**,**p**) Comparison of EoI and artifact marks. For the EEG signal on the left (**a**), the core SNN indicates an EoI by eliciting spikes (**d**). The absence of activity from the artifact detection SNN (no spikes in g) indicates that no artifact occurred at this time. (**h**) The EEG SNN classifies this EoI as HFO. The EEG signal on the right (**i**) elicits spikes in the core SNN, which indicate an EoI (**l**). The activity from the neurons in the artifact detection SNN (**o**) indicates that an artifact occurred at this time. (**p**) The EEG SNN marks this EoI as an artifact.

We used the spikes that marked an artifact to decide whether an EoI should be classified as an HFO. This step was performed outside of the SNN. The 15 ms time window after a spike was taken as the duration of an artifact. Any EoI overlapping with an artifact was rejected (Fig. 2p,h).

### Rejection of HFO in the contralateral channel

Each EEG SNN analyzed the signal of a bipolar EEG channel. To further discard false HFO detections, we eliminated HFO that occurred simultaneously in the homologous channel of the other hemisphere. This elimination was done outside of the EEG SNN. We compared the time points where the EEG SNN detected an HFO in the signals from a channel and its contralateral channel and eliminated the HFO where the two markings overlapped.

### Example of a detected HFO and a sharp transient artifact > 500 Hz

Figure 2 shows the activity in the core SNN and the artifact detection SNN in response to an exemplary HFO and an exemplary artifact.

As an example for an HFO, Fig. 2d shows the raster plot of the second layer neurons in the core SNN. Four of these neurons elicited spikes in response to the signal shown in Fig. 2a, which indicated the presence of an EoI. Figure 2g shows the raster plot of the second layer neurons in the artifact detection SNN in response to the same signal. None of these neurons responded, meaning that no artifact was detected. Hence, this EoI (Fig. 2h: green line) was classified as HFO (Fig. 2h: purple line).

As an example for an artifact, Fig. 2l shows the raster plot of the second layer neurons in the core SNN. In this case, 48 of these neurons elicited spikes in response to the signal in Fig. 2i, which also indicated the presence of an EoI. However, on this occasion, three of the neurons in the artifact detection SNN elicited a response (Fig. 2o), indicating the presence of an artifact in the signal (Fig. 2p: yellow line). Hence, this EoI (Fig. 2p: green line) was not classified as HFO but as an artifact.

### HFO rates from the SNN and the spectrum detector are comparable

We next determined the HFO rate. For each patient, we counted the number of HFO detected per electrode channel and divided this number by the recording duration (mean 27 min, total data duration 544 min) to obtain the HFO rate (Table 2). We found HFO rates ≥ 0.25 HFO/min in the recordings of patients with active epilepsy (100 recording intervals, median duration 4.5 min, median rate 1.4 HFO/min, range [0.11–8.78] HFO/min). The HFO rates of the Spectrum and the SNN detector were correlated across all recordings (rho = 0.83 CI [0.61 0.93], *p* < 0.0001, Spearman’s rank correlation). In total, the SNN and the Spectrum detector found 6275 HFOs and 5249 HFOs in the affected hemisphere, respectively.

**Table 2:** Patient characteristics at the pre- and postsurgical EEG recordings.

Patient features			Presurgical EEG recordings			Epilepsy surgery			Postsurgical EEG recordings		
No	Age, sex	Etiology	Seizure frequency (seizures/month	HFO rate (HFO/min)		Resected area	Follow-up period (months)	ILAE outcome	Seizure frequency (seizures/month	HFO rate (HFO/min)	
				Affected hemisphere	Non-affected hemisphere					Affected hemisphere	Non-affected hemisphere
1	4, f	Sturge Weber syndrome	30	6.38	6.88	R lateral posterior temporal and lateral occipital	51	3	0.2	0.13	0.01
2	5, m	FCD 1a	180	5.6	0.54	L medial/lateral anterior temporal	27	5	180	2.05	1.28
2	7, m	FCD 1a	180	1.93	1.26	L temporo-posterior, occipital, parietal	67	1	0	0.09	0.09
3	10, m	diffuse astrocytoma	0.5	0.66	0.73	R medial anterior temporal	45	3	1	0.79	0.76
4	3, m	mMCD	2	0.24	1.1	R medial/lateral anterior temporal	45	5	150	4.1	5.09
5	13, m	cavernoma	8	0.88	0.07	L dorsal medial/lateral prefrontal	35	1	0	0.02	0
6	15, m	DNET	12	0.11	0.22	R medial/lateral anterior temporal	11	1	0	0.03	0.01
7	14, f	ganglioglioma	4	0.85	0.15	L inferior/basal temporal	33	1	0	0.05	0.13
8	1, f	polymicrogyria, FCD 1a	450	8.78	2.39	R dorsal lateral prefrontal	51	5	4	n/a	n/a
9	3, m	FCD 2a	30	n/a	n/a	R dorsal lateral prefrontal	75	1	0	0.1	0.1
10	6, f	angiocentric glioma	0.5	n/a	n/a	R dorsal lateral prefrontal	60	5	0.3	0.11	0.07
11	17, m	perinatal ischemic lesion	2	n/a	n/a	R lateral posterior temporal & lateral occipital	38	1	0	0.17	0.53

Seizure etiology, pre- and postsurgical seizure frequency, lateralization and localization of surgical resection, postsurgical follow-up duration, final seizure outcome according to the ILAE classification, HFO recording channels and rates in the pre- and postsurgical EEG. m: male; f: female; L: left; R: right; FCD: focal cortical dysplasia; mMCD: mild malformation of cortical development; DNET: Dysembryoplastic neuroepithelial tumor; ECoG: electrocorticography; HFO: high frequency oscillations.

### Comparison of HFO rates between hemispheres

The SNN found a higher HFO rate in the affected than in the non-affected hemisphere in only 9 of all 14 recordings in patients with active epilepsy. The HFO rate was lower in the affected than in the non-affected hemisphere for the remaining five recordings. Three of these five recordings were in patients with deep lesions (Patients 3 and 4). In these two patients, the median HFO rate was 0.57 HFO/min and thus considerably lower than the median HFO rate in the presurgical recordings of patients with more superficial frontal, temporal or occipital lesions (0.97 HFO/min). Across all 14 recordings in patients with active epilepsy, the HFO rates did not differ significantly between the affected and the non-affected hemisphere (*p* = 0.3, Wilcoxon matched-pairs signed-rank test).

### HFO rate mirrors epilepsy severity

**Figure 3:**
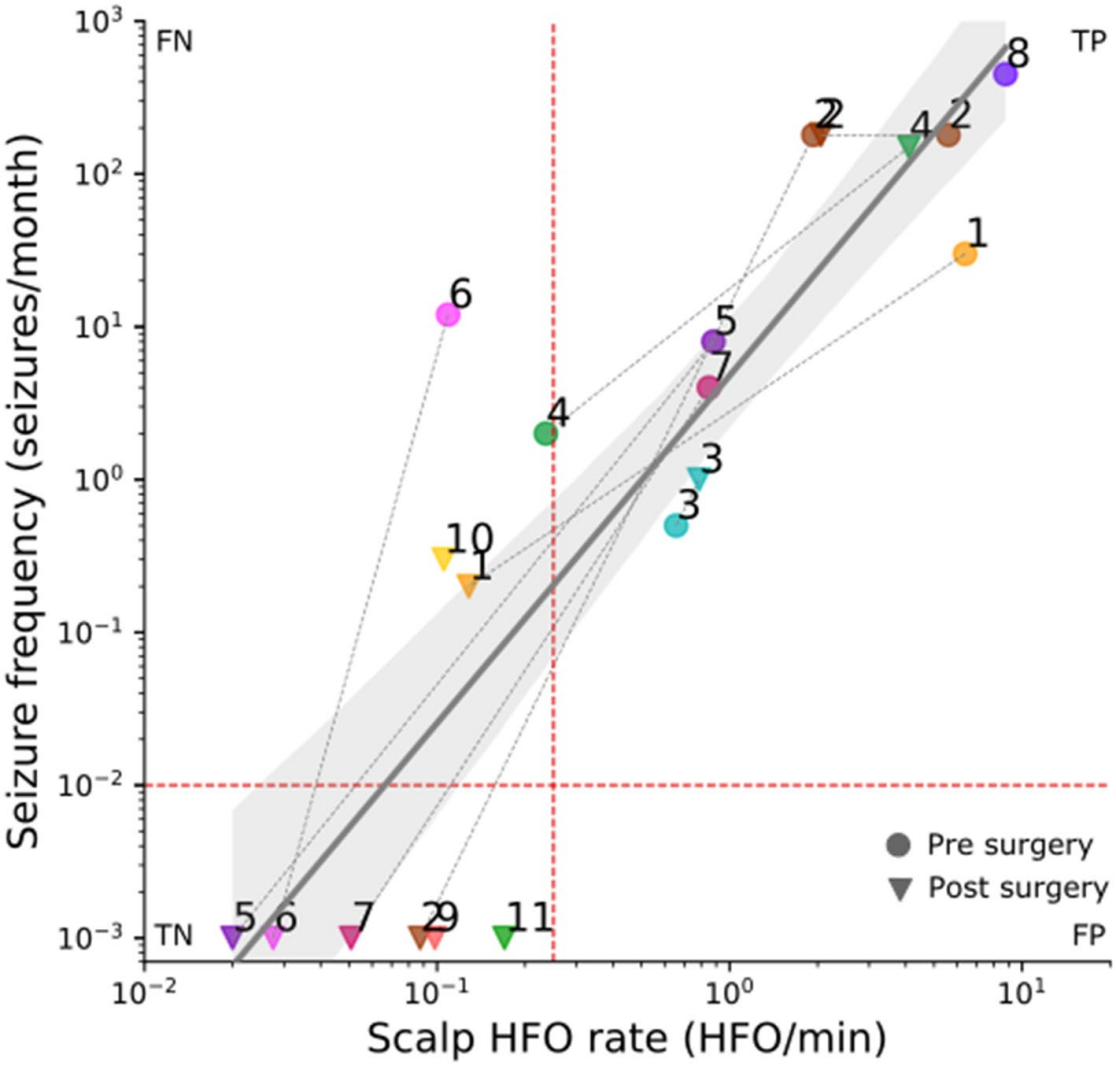
HFO rate mirrors seizure frequency. EEG recordings before (circles) and after (triangles) epilepsy surgery were pooled across our patient cohort. Each point in the plot corresponds to an EEG recording and indicates the seizure frequency and HFO rate at the time of the EEG recording. Axes are on a logarithmic scale. For illustrative purpose, seizure freedom was approximated by 10^–3^ seizures/month so that only a seizure frequency > 10^–2^ seizures/month indicates active epilepsy (horizontal red line). If the HFO rate exceeded the rate threshold of 0.25 HFO/min (vertical red line), the recording was defined as showing HFO. The recordings from ten patients with active epilepsy were above this threshold (TP = 10), while the recordings from four patients with active epilepsy were below this threshold (FN = 4). After their surgery, patients 2,5,6,7,9,11 were seizure-free, and their HFO rate was below the threshold (TN = 6). The HFO rate in the scalp EEG correlated with seizure frequency (R^2^ = 0.76, *p* < 0.0001, linear regression; rho = 0.90, *p* < 0.0001, Spearman’s correlation). TP True Positive; TN True Negative; FP False Positive; FN False Negative.

We first investigated the association between HFO rate and epilepsy severity across all recordings in a cross-sectional approach. Table 2 and Fig. 3 show the seizure frequencies of our patients and the HFO rate at each recording, before and after surgery. HFO rates over the affected hemisphere exceeded the threshold of 0.25 HFO/min in ten EEG recordings, all from patients with active epilepsy (TP = 10, PPV = 100%) and was below this threshold in ten recordings, six of which were from seizure-free patients (TN = 6, FN = 4, NPV = 60%, sensitivity = 71%). The threshold was not exceeded in any of the recordings from seizure-free patients (FP = 0, specificity = 100%). Hence, the SNN associated the HFO rate with active epilepsy with an accuracy of 80% CI [56% 94%] (Table 3). This finding is in line with the prediction of the Spectrum detector in the same dataset (85% accuracy^14^). Similarly, HFO rates were higher in patients with active epilepsy (20 recordings, *p* = 0.003, Wilcoxon rank-sum test).

**Table 3:** The occurrence of HFO is associated with epilepsy state.

	Spectrum detector prediction [%]	SNN prediction [%]
Specificity = TN/(TN + FP)	67	100
Sensitivity = TP/(TP + FN)	93	71
Negative predictive value = TN/(TN + FN)	80	60
Positive predictive value = TP/(TP + FP)	87	100
Accuracy = (TP + TN)/N [%]	85	80

Comparison of epilepsy state prediction (active epilepsy: seizures/month > 0) between the Spectrum detector and the SNN. TP True Positive; TN True Negative; FP False Positive; FN False Negative; N = TP + TN + FP + FN = number of patients.

As a further result, illustrated in Fig. 3, HFO rate over the affected hemisphere correlated with seizure frequency across all recordings (rho = 0.90 CI [0.75 0.96], *p* < 0.0001, Spearman’s correlation). We next performed a linear regression to estimate whether the HFO rate predicts the seizure frequency. The equation for the regression of seizure frequency on HFO rate becomes log_10_(seizure_frequency) = 2.27 * log_10_(HFO_rate) + 0.7 with R^2^ = 0.76 CI [0.61 0.91], *p* < 0.0001. This means that, on average, the HFO_rate = 2 HFO/min corresponds to seizure_frequency = 25 seizures/month. A reduction to 1 HFO/min would predict a reduced seizure_frequency = 5 seizures/month.

### HFO rate reflects the response to surgical therapy

**Figure 4:**
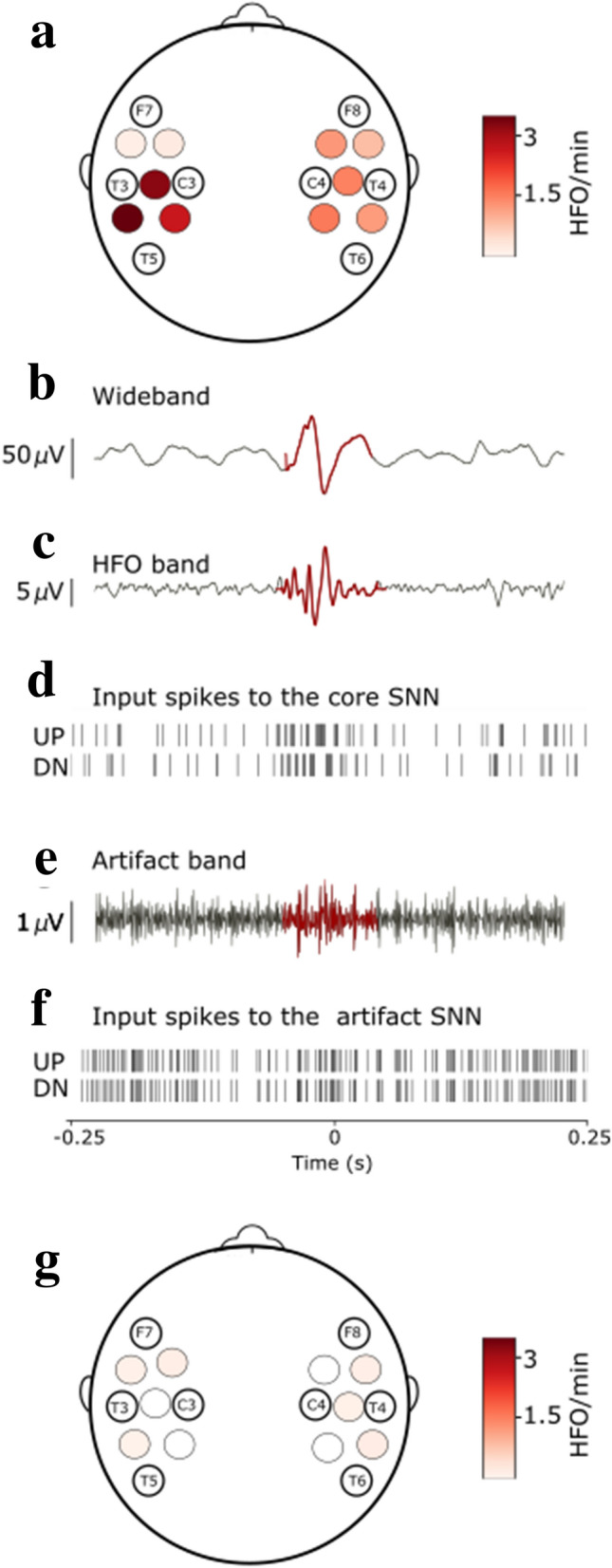
HFO rates in the pre- and postsurgical EEG of Patient 2. HFO rates and their localization in pre- (**a**) and postsurgical (**g**) EEG after the second surgery of Patient 2. Example of an HFO detected by the EEG SNN in the presurgical EEG in the bipolar channel T3-T5 (**b**–**f**). We filtered the wideband signal (**b**) in the ripple band (80–250 Hz) (**c**) and converted it to spikes (**d**). These spikes are the input to the core SNN for EoI detection. (**e**) We filtered the same signal in the 500–900 Hz band and converted it to spikes (**f**). These spikes are the input to the artifact detection SNN for artifact detection. Patient 2 underwent resective epilepsy surgery within the left hemisphere and achieved seizure freedom (follow-up 67 months). The EEG SNN found a 3.5 HFO/min maximum mean HFO rate in the presurgical and 0.2 HFO/min in the postsurgical recordings. The decrease of HFO rate after surgery in this patient mirrored the decrease in seizure frequency from 180 seizures/month before surgery to postsurgical seizure freedom.

Next, in a longitudinal approach, we investigated whether a change in seizure frequency (before and after surgery) entails a change in HFO rate. Figure 4 illustrates the HFO rate, as recorded before and after surgery, in the example of the second surgery of Patient 2. This patient underwent a left temporo-parieto-occipital second surgery and achieved seizure freedom. The decrease of the HFO rate after surgery in this patient mirrored the decrease in seizure frequency from 180 seizures/month before surgery to seizure freedom after surgery.

However, in Patient 2 it should be noted that the scalp HFO rate over the affected hemisphere remained particularly high following his first surgery, corresponding to the patient's disease state which remained unaffected by this first surgery. In this case, there is no clear-cut correlation between the relative pre-post-1st-surgery decrease of HFO rate and the unchanged seizure frequency. This divergence from the overall finding that HFO rate mirrors seizure frequency may at least partly be attributed to the fact that the reliability of both seizure frequency and HFO rate measurements may be prone to some uncertainty.

Still, HFO rate reflected the response to surgical therapy in the majority of patients (Fig. 3). The decrease in HFO rate over the affected hemisphere corresponded to a decrease in seizure frequency following the full resection of the epileptogenic zone in Patients 5, 6, 7, and Patient 2 (second surgery) (0.88 to 0.02 HFO/min, 0.11 to 0.03 HFO/min, 0.85 to 0.05 HFO/min, and 1.93 to 0.09 HFO/min, respectively), and the partial resection of the epileptogenic zone in Patient 1 (6.38 to 0.13 HFO/min). The increase in HFO rate (0.66 to 0.79 HFO/min and 0.24 to 4.1 HFO/min) reflected an increase in seizure frequency in Patients 3 and 4 (0.5 to 1 seizure/month and 2 to 150 seizures/month). Patient 4 failed to respond to surgery due to an underlying genetic disorder. Postsurgical HFO rate was particularly low (0.02, 0.03, 0.05, 0.1, 0.17, and 0.09 HFO/min) in all patients that achieved seizure freedom (Patient 5, 6, 7, 9, 11, and Patient 2 surgery 2) and particularly high (2.05 and 4.1 HFO/min) in the two patients that remained unaffected by surgery (Patient 2 surgery 1, and Patient 4). Intra-individual decrease in HFO rate between pre- and postsurgical recordings mirrored decrease in seizure frequency (8 cases, χ^2^_1_ = 8, p = 0.0047).

## Discussion

We devised a simulated novel SNN that can automatically detect and correctly distinguish EoI in scalp EEG as artifacts or HFO. The occurrence of HFO was associated with active epilepsy with 80% accuracy. Across all patients and EEG recordings, the HFO rate correlated with seizure frequency. In individual patients, the HFO rate mirrored the decrease or increase in seizure frequency after surgery. We thereby demonstrated the feasibility of HFO detection in scalp EEG with a neuromorphic SNN.

### Comparison with the Spectrum detector

The HFO rates measured by our EEG SNN correlated with the rates detected by the well-established and clinically validated Spectrum detector, as applied to the same EEG dataset in our previous work^14^. In designing the EEG SNN, we did not aim for one-to-one agreement with the Spectrum detector on the detected HFO events. Instead, we aimed to prove that the HFO rate can be used to determine our patients' epilepsy severity and seizure frequency. Both detectors reached high accuracy in classifying epilepsy severity across all recordings (Spectrum 85%, SNN 80%). However, the two detectors did not agree on the classification of each patient. Furthermore, both detectors established a significant correlation of HFO rate with seizure frequency.

### Comparison between hemispheres

While the Spectrum detector found a significantly higher HFO rate in the affected than in the non-affected hemisphere (*p* = 0.0003)^14^, this was not the case for the SNN (*p* = 0.3). The discrepancy between detectors is most apparent in the HFO rates of the patients with a deep-seated lesion, i.e., where the recording EEG channels were located far from the HFO generator. In these patients, both detectors found lower HFO rates than in patients with more superficial lesions, suggesting a lower signal-to-noise ratio. This observation suggests that the SNN may be more prone to the low signal-to-noise ratio than the Spectrum detector.

### Cohort size

The present study was conducted in a small cohort that is highly heterogeneous with respect to age and underlying etiology (Table 2). In a proof-of-concept approach, we showed that the SNN detector in the present study performs similarly well to the Spectrum detector in our previous study on the same data^14^. Of note, despite the small and heterogeneous cohort of this study, the CI was above chance level (50%) regarding classification accuracy 80% CI [56% 94%]. However, larger cohorts will be required to robustly claim the clinical significance of the proposed HFO detection technology.

### SNN features

The core SNN used here has been previously shown to detect clinically relevant HFO in the presurgical long-term iEEG^8^ and the intraoperative ECoG^32^ and has been adapted here for HFO detection in the scalp EEG. Our approach for HFO detection using the SNN exploits the simulated temporal dynamics of neurons and synapses to determine the optimal detection parameters compatible with analog circuit distributions. This approach to detecting HFO differs considerably from the one followed in deep neural network algorithms, which usually perform very well. However, they are sample-inefficient (i.e., they require ample computation time) and thus, are unsuitable for implementation in a wearable device. In contrast, the SNN employs a shallow network with carefully tuned weights and time constants, enabling its future implementation in neuromorphic hardware.

### Future implementation in neuromorphic hardware

Our simulated EEG SNN has been motivated by the perspective of future implementation in neuromorphic processors that carry out computation “at the edge''^47–49^. The EEG SNN can be easily mapped onto the neuromorphic device that we have developed and described previously^8^. All parameters and architecture elements in this neuromorphic device have been carefully chosen to enable the implementation of the simulated EEG SNN in the neuromorphic hardware with only minor adaptations.

A hardware HFO detector based on neuromorphic technology would benefit from low power consumption since it performs spike-based processing. The raw signal is converted into "events" by an asynchronous delta modulator (ADM) circuit. There is no fixed sampling rate. The event rate depends only on the amplitude and slope of the signal. Since HFO are sparse, the signal acquisition stage is very low-power. Similarly, the mixed-signal analog/digital SNN circuits are data-driven: they are activated and consume power only if and when events are arriving from the ADM. This feature makes the whole device highly efficient in terms of power consumption. As an output, only the presence of an HFO would be signaled to a data storage device, e.g., a mobile phone.

To estimate power consumption, we envision a real-time HFO analysis “at the edge” that includes signal amplification in the preprocessing stage, HFO detection in the SNN, and wireless transmission of a flag to a storage device at the time of HFO occurrence. As previously reported^8^, our chip consumes 58.4 μW for preprocessing and 555.6 μW for the SNN. The flag is conveyed by a miniature bluetooth low-energy transmitter (8.6 × 3.3 × 0.9 mm including the antenna) with 25.2 μW per channel, which amounts to about 20% of the total power consumption. Using a standard battery with 660 mAh capacity and weighing 1.8 g, this device would have the capacity to operate continuously for over 12 days. Thus, our neuromorphic processor's in-memory computing spike-based processing would result in a compact and battery-powered device. These characteristics support our use-case of long-term EEG recordings for non-invasive epilepsy treatment monitoring.

### Outlook on ultra-long-term epilepsy monitoring

Clinical assessment would greatly benefit from long-term epilepsy monitoring by a wearable device. Wearable devices for out-of-hospital epilepsy monitoring over months or years represent a potential breakthrough in epilepsy diagnosis and treatment, as they may facilitate more accurate seizure detection and thus enable the delivery of therapies with increased efficacy and fewer side effects^26–28^. Most current commercial devices aim for seizure detection and use non-EEG signals. However, they currently suffer from high false alarm rates; only five current wearable devices perform satisfactorily in phase III studies^26^.

The analysis of scalp EEG signals, among them HFO, may improve epilepsy monitoring. Ultra-long-term recordings of scalp EEG and subcutaneous EEG lasting several months were performed in a research setting^50,51^. They found seizures to occur in cyclical recurrence and showed that around 50% of all seizures remained unnoticed by patients and proxies (comparison of seizure diaries and detected seizures). Given the correlation between HFO rate and seizure frequency (Fig. 3), the detection of HFO from scalp EEG recordings may improve the monitoring of epilepsy severity in clinical practice.

## Conclusion

The automated SNN detector ensures a prospective, bias-free definition of HFO in scalp EEG. The HFO rate mirrors seizure frequency, and thus epilepsy severity, in pediatric drug-resistant focal epilepsy. We view this finding in a small and heterogeneous cohort as the first proof-of-principle for the clinical relevance of the HFO detected with the SNN. Being compatible with neuromorphic technology, the implementation of the EEG SNN in a neuromorphic device might provide a further step towards non-invasive therapy monitoring in patients affected by epilepsy.

## Data Availability

The scalp EEG data and the code for the SNN are freely available at our website https://hfozuri.ch/. The scalp EEG data with the HFO markings by the Spectrum detector are freely available at https://gin.g-node.org/USZ_NCH/Scalp_EEG_HFO, and the SNN detector can be found at https://github.com/kburel/snn-hfo-detection.
